# Point-Of-Care CAR T-Cell Production (ARI-0001) Using a Closed Semi-automatic Bioreactor: Experience From an Academic Phase I Clinical Trial

**DOI:** 10.3389/fimmu.2020.00482

**Published:** 2020-03-20

**Authors:** Maria Castella, Miguel Caballero-Baños, Valentín Ortiz-Maldonado, Europa Azucena González-Navarro, Guillermo Suñé, Asier Antoñana-Vidósola, Anna Boronat, Berta Marzal, Lucía Millán, Beatriz Martín-Antonio, Joan Cid, Miquel Lozano, Enric García, Jaime Tabera, Esteve Trias, Unai Perpiña, Josep Ma Canals, Tycho Baumann, Daniel Benítez-Ribas, Elías Campo, Jordi Yagüe, Álvaro Urbano-Ispizua, Susana Rives, Julio Delgado, Manel Juan

**Affiliations:** ^1^Department of Hematology, Institut Clínic de Malalties Hematològiques i Oncològiques, Hospital Clínic de Barcelona, Barcelona, Spain; ^2^Institut D'Investigacions Biomèdiques August Pi i Sunyer (IDIBAPS), Barcelona, Spain; ^3^Blood and Tissue Bank (BST), Barcelona, Spain; ^4^Department of Immunology, Centro de Diagnóstico Biomédico, Hospital Clínic de Barcelona, Barcelona, Spain; ^5^Hospital Sant Joan de Déu, Barcelona, Spain; ^6^Department of Hemotherapy and Hemostasis, Institut Clínic de Malalties Hematològiques i Oncològiques, Hospital Clínic de Barcelona, Barcelona, Spain; ^7^Apheresis Unit, Hospital Sant Joan de Déu de Barcelona, Barcelona, Spain; ^8^Unit of Advanced Therapies, Hospital Clínic de Barcelona, Barcelona, Spain; ^9^Stem Cells and Regenerative Medicine Laboratory, Department of Biomedical Sciences, Production and Validation Center of Advanced Therapies (Creatio), Universitat de Barcelona, Barcelona, Spain; ^10^Universitat de Barcelona, Barcelona, Spain; ^11^Department of Pathology, Hospital Clínic de Barcelona, IDIBAPS, Barcelona, Spain; ^12^Centro de Investigación Biomedical en Red de Cancer, Barcelona, Spain; ^13^Institució Catalana de Recerca i Estudis Avancats, Barcelona, Spain; ^14^Department of Biomedicine, School of Medicine, Josep Carreras Leukemia Research Institute, Universitat de Barcelona, Barcelona, Spain; ^15^Immunotherapy Unit Blood and Tissue Bank-Hospital Clínic de Barcelona, Barcelona, Spain; ^16^Department of Pediatric Hematology and Oncology, Hospital Sant Joan de Déu, Barcelona, Spain; ^17^Institut de Recerca Sant Joan de Déu, Barcelona, Spain

**Keywords:** chimeric antigen receptor, CD19, leukemia, lymphoma, immunotherapy, CAR T-cell production, CliniMACS Prodigy

## Abstract

Development of semi-automated devices that can reduce the hands-on time and standardize the production of clinical-grade CAR T-cells, such as CliniMACS Prodigy from Miltenyi, is key to facilitate the development of CAR T-cell therapies, especially in academic institutions. However, the feasibility of manufacturing CAR T-cell products from heavily pre-treated patients with this system has not been demonstrated yet. Here we report and characterize the production of 28 CAR T-cell products in the context of a phase I clinical trial for CD19+ B-cell malignancies (NCT03144583). The system includes CD4-CD8 cell selection, lentiviral transduction and T-cell expansion using IL-7/IL-15. Twenty-seven out of 28 CAR T-cell products manufactured met the full list of specifications and were considered valid products. *Ex vivo* cell expansion lasted an average of 8.5 days and had a mean transduction rate of 30.6 ± 13.44%. All products obtained presented cytotoxic activity against CD19+ cells and were proficient in the secretion of pro-inflammatory cytokines. Expansion kinetics was slower in patient's cells compared to healthy donor's cells. However, product potency was comparable. CAR T-cell subset phenotype was highly variable among patients and largely determined by the initial product. T_CM_ and T_EM_ were the predominant T-cell phenotypes obtained. 38.7% of CAR T-cells obtained presented a T_N_ or T_CM_ phenotype, in average, which are the subsets capable of establishing a long-lasting T-cell memory in patients. An in-depth analysis to identify individual factors contributing to the optimal T-cell phenotype revealed that *ex vivo* cell expansion leads to reduced numbers of T_N_, T_SCM_, and T_EFF_ cells, while T_CM_ cells increase, both due to cell expansion and CAR-expression. Overall, our results show for the first time that clinical-grade production of CAR T-cells for heavily pre-treated patients using CliniMACS Prodigy system is feasible, and that the obtained products meet the current quality standards of the field. Reduced *ex vivo* expansion may yield CAR T-cell products with increased persistence *in vivo*.

## Introduction

Adoptive T-cell transfer (ACT) immunotherapy is a field in continuous expansion especially during the last three decades. ACT involves *ex vivo* expansion of tumor-specific cells and reinfusion into the patient. Among these therapies, the use of Chimeric Antigen Receptor (CAR) T-cells for the treatment of several hematologic malignancies has shown unprecedented efficacy rates. Consequently, the development of these therapies from bench to bedside has been done in an impressively short amount of time (1–3).

CAR T-cells are genetically modified cells whose cytotoxic activity has been redirected toward cancer cells with the aim of eliminating the tumor. CARs are chimeric proteins composed of an extracellular region responsible for binding to a particular antigen and an intracellular region that promotes T-cell cytotoxic activity and proliferation. CAR binding to the selected antigen is usually mediated by a single chain variable fragment (scFv) of a monoclonal antibody. The scFv-derived region results in a MHC-independent interaction of the CAR with its ligand. This scFv is combined with one or more intracellular co-stimulatory domains (usually CD28 or 4-1BB) and a pro-activator cytotoxic domain (CD3ζ) (4–6).

First great successes in the CAR T-cell field came from studies using a 2nd generation CAR (including a co-stimulatory domain) targeting the pan-B-cell lineage marker CD19. In this studies by National Cancer Institute and University of Pennsylvania, one patient with follicular lymphoma experienced partial remission (7) and two out of three patients with advanced refractory chronic lymphocytic leukemia (CLL) obtained complete responses (8). Afterwards, these and several other institutions including Memorial Sloan Kettering Cancer Center and the Fred Hutchinson Cancer Research Center pioneered several studies using slightly different CAR19 constructs, and confirmed response rates between 50 and 80% in CLL, non-Hodgkin's lymphoma (NHL) and acute lymphoblastic leukemia (ALL) (9–14).

Currently, a lot of effort is being put in developing new CARs to target other types of tumors. Anti-BCMA CAR has also shown impressive response rates (around 90%) for multiple myeloma (MM) (15–17). Also, other antigens such as CD30 and CD22 are currently being explored for the treatment of hematologic malignancies (18–20). In the field of solid tumors, CAR T-cell therapies have proven to be more challenging, so far. Initial clinical trials in solid tumors have shown limited efficacy and high toxicity (21–30). This fact can be attributed to several factors: difficulty in finding tumor-specific antigens to target, poor T cell infiltration in the tumor and immunosuppressive tumor microenvironment, among others. The scientific community is currently working in finding ways to overcome these challenges (31, 32). As a result, dozens of clinical trials using 4th generation CARs (Armored CARs, CAR T-cells containing suicide genes and antibody-producing CARTs) for solid tumors are ongoing (33–36).

The different clinical applications and the number of patients waiting to receive a treatment are exponentially growing. Accordingly, the number of institutions and centers that are in need of being capable of performing CAR T-cell therapies is also growing. In this context, developing systems that can facilitate CAR T-cell production is crucial to help CAR T-cell field move faster, thereby finding effective therapies for all kinds of malignances and other diseases.

To this end, we have previously reported the development and pre-clinical evaluation of a new anti-CD19 CAR, based on the A3B1 antibody (37). Concomitantly, we have also established a CAR T-cell (ARI-0001) production system at our institution (Hospital Clinic de Barcelona). Our system is based on the use of a closed semi-automatic bioreactor (CliniMACS Prodigy®) for *ex vivo* selection, transduction and expansion of CAR T-cells. We are currently conducting a phase I clinical trial using our novel anti-CD19 CAR for CD19+ B-cell malignancies (NCT03144583). We have successfully prepared 28 ARI-0001 cell products in the context of this clinical trial. Here, we present the results and characteristics of the products obtained, thereby demonstrating, for the first time, the feasibility of CAR T-cell production in a relatively wide series of heavily pre-treated patients using CliniMACS Prodigy system.

## Materials and Methods

### Patients and Samples

At the time of submitting this manuscript, 28 products from 27 patients enrolled in phase I clinical trial for CD19+ B-cell malignancies (NCT03144583) have been produced. Among the 27 patients, 22 had ALL (14 adult and eight pediatric patients), four had NHL and 1 CLL. All patients included in the clinical trial had relapsed of their disease. Patients' pretreatment regimens are summarized in Table 1.

**Table 1:** Patients' pretreatment regimens.

**Pat ID**	**Age**	**Sex**	**Disease**	**Lines of treatment prior to leukoapheresis**	**Allogeneic HCT**
T01	27	M	ALL	PETHEMA 2011, blinatumomab, radiotherapy	Yes (+ DLI)
T02	25	M	PMLBCL	R-CHOP, R-ESHAP, autologous HCT, radiotherapy, brentuximab, GSK525762, nivolumab	No
T03	7	F	ALL	SEHOP 2013, SEHOP 2016, inotuzumab, methotrexate + cyclophosphamide + anthracyclins + prednisone	Yes (+ DLI)
T04	19	F	PMLBCL	R-CHOP, R-ESHAP, autologous HCT, radiotherapy, BURKIMAB, Gemcitabine + vinorelbine + procarbacine	No
T05	51	M	DLBCL	BURKIMAB, autologous HCT, cyclophosphamide + prednisone, GSK525762, methotrexate, cyclophosphamide	No
T06	20	F	ALL	PETHEMA 2011, PETHEMA 2008	Yes
T07	19	M	ALL	PETHEMA 2011, FLAG-Ida	Yes
T08	53	F	CLL	FCR, BR, ibrutinib, venetoclax, obinutuzumab, idelalisib	No
T09	8	M	ALL	SEHOP 2008, SEHOP 2013	No
T13	20	M	ALL	GRAAL 2003, FLAG-Ida, blinatumomab	Yes (x2)
T11	34	F	ALL	PETHEMA 2011, Hyper-CVAD, inotuzumab	Yes
T12	3	F	ALL	SEHOP 2013, SEHOP 2016, vincristine + prednisone	Yes
T14	27	M	ALL	PETHEMA 2008, FLAG-Ida, PETHEMA 2011	Yes
T15	30	M	ALL	PETHEMA 2011, FLAG-Ida, FLAG-Ida + blinatumomab, FLAG-Ida, inotuzumab	Yes (x2 + DLI)
T16	10	M	ALL	SEHOP 2013	Yes
T17	23	M	ALL	PETHEMA 2011, FLAG-Ida, PETHEMA 2008, BFM-90, inotuzumab	Yes
T19	9	F	ALL	PETHEMA, SEHOP 2015, radiotherapy	No
T20	35	M	ALL	PETHEMA 2011, FLAG-Ida, PETHEMA 2011, methotrexate + vincristine + dexamethasone	Yes
T21	13	F	ALL	SEHOP 2013, SEHOP 2016, prednisone	Yes
T22	29	M	ALL	PETHEMA 2008, dasatinib, FLAG-Ida + ponatinib, FCR	Yes
T24	19	M	ALL	PETHEMA 2011, FLAG-Ida	No
T25	47	F	ALL	PETHEMA 2011, FLAG-Ida, PETHEMA 2011	Yes
T26	8	F	ALL	SEHOP	Yes
T27	22	M	ALL	PETHEMA 2008, FLAG-Ida, PETHEMA 2011	No
T30	31	M	ALL	PETHEMA 2008, blinatumomab	Yes (+DLI)
T32	23	M	ALL	PETHEMA 2008, PETHEMA 2011, vindesine + prednisone, inotuzumab	Yes (x2)
T34	45	F	DLBCL	R-CHOP, R-ESHAP, BURKIMAB, radiotherapy	No

*ALL, acute lymphoblastic leukemia; DLBCL, diffuse large B-cell lymphoma; PMLBCL, primary mediastinal large B-cell lymphoma; CLL, chronic lymphocytic leukemia; DLI, donor lymphocyte infusion; HCT, hematopoietic cell transplantation; FCR, fludarabina + cyclophosphamide + rituximab; BR, bendamustina + rituximab; FLAG-Ida, fludarabina + cytarabine + idarubicin + G-CSF; PETHEMA, Spanish Program of Treatments in Hematology; SEHOP, Spanish Society of Pediatric Hematology & Oncology; GRAAL, Group for Research on Adult Acute Lymphoblastic Leukemia*.

Adult patients were subjected to leukocytapheresis at the Apheresis Unit, Hospital Clínic, and pediatric patients at the Apheresis Unit of Hospital Sant Joan de Déu/BST, after signing an informed consent. Apheresis procedures were performed using Amicus device (Fresenius Kabi, Lake Zurich, IL). A minimum of 1 × 10^8^ total T-cells diluted in 50 ml of plasma were required. This study has been approved by the Research Ethics Comitee (CeIm) of Hospital Clinic. HCB/2017/0001. Clinical trial: CART19-BE-01. Eudra: 2016-002972-29.

### ARI-0001 Cell Production

Apheresis products were connected to CliniMACS Prodigy® system (Miltenyi Biotec) tubing set. Erythrocytes and platelets were removed by density gradient centrifugation in the Centricult unit. The remaining cells were selected using CD4 and CD8 coated magnetic beads. Selected cells were eluted in the “Reapplication Bag.” After selection, 1 × 10^8^ T-cells (from reapplication bag) were used to initiate cell culture. The remaining cells were cryopreserved in bags and vials to be used as control cells for product quality assays and as a backup in case of production failure. Cells were cultured using TexMACS® media supplemented with 3% human AB serum (obtained from the blood bank. BST) and with 155 IU/mL IL-7 and 290 IU/mL IL-15 (Miltenyi Biotec #170-076-111 and #170-076-114, respectively). Cells were immediately activated using TransACT GMP Grade (Miltenyi Biotec, Cat. N. 170-076-156) and transduced 24h later using CAR19-containing lentivirus at MOI = 10. A cell culture wash was programmed 48 h after transduction. The cells were then maintained in culture with increasing shaking until the desired cell number was reached (typically 7–10 days after cell culture initiation). Cells were finally eluted in 100 ml 0.9% NaCl + 1%HSA, aliquoted according to the desired ARI-0001 cell dose and cryopreserved until infusion.

The aim was to achieve two doses of ARI-0001 cells/patient. The planned target cell dose varied depending on the patient's disease. Typically, 1 × 10^6^ ARI-0001 cells/kg for patients with ALL and CLL, and 5 × 10^6^ ARI-0001 cells/kg for NHL patients.

### Monoclonal Antibodies

CAR19 expression was detected with an APC-conjugated AffiniPureF(ab')_2_-fragment goat-anti-mouse IgG (Jackson ImmunoResearch Laboratories, 115-136-072). ARI-0001 cell product composition was determined by flow cytometry using staining with the following antibodies (all from BD): CD45-APC, CD3-BV421, CD4-FITC, CD8-PerCPCy5.5, CD19-PECy7, CD16-PE, CD56-PE.

For the T cell subset characterization experiments, CAR+ cells were detected using a CD19-Fc recombinant protein chimera (R&D, Cat. N. 9269-CD-050) and a secondary antibody FITC-Goat F(ab)_2_ anti-human IgG (Life Technologies, Cat. N. H10101C). This staining was combined with the following monoclonal antibodies (all from BD): CD3-BV421, CD8-APC.Cy7, CD45RA-PECy7, CD45RO-APC, CCR7-PerCPCy5.5, CD28-BV510, and CD95-PE (or CD27-PE). T cell subpopulations were defined as follows: T_N_: CD45RA+, CCR7+; T_SCM_: CD45RA+, CCR7+,CD95+; T_CM_: CD45RA–, CCR7+; T_EM_: CD45RA-, CCR7- and T_EFF_: CD45RA+, CCR7−.

For intracellular cytokine measurement, the following antibodies were used, all from BD: CD3-BV450, CD8-APC.H7, CD4-BV500, IFNγ-PerCP.Cy5.5, TNFα-PE.

For repeated challenges experiment, the antibodies used were the following, all from BD: CD3-APC, CD4-BV510, CD8-APC.Cy7, CD19-PE.

For flow cytometry analyses, cells were acquired using a FACS Canto II, BD and subsequently analyzed using FlowJo Software.

### Product Quality Controls

Product potency assay was performed by flow cytometry as described in Castella et al. (37). Real-time PCR was used to measure number of copies/cell and to assess the presence of replication-competent lentivirus (RCL) in the final product. Primer sequences and PCR protocol has been described previously (37). Product sterility, absence of mycoplasma, endotoxin and adventitious virus was determined by a certified laboratory using the technique specified in Table S1. Adventitious virus included the determination of HIV virus presence among others. Since conventional HIV detection methods detect also the presence of the lentiviral transgene used to transduce the cells, an alternative PCR assay based on the detection of Env gene was used to discriminate between HIV infection and lentiviral transduction. The primers used to amplify Env gene are: Env_F: 5′CAATGTACACATGGAATTAGGCCA 3′ and Env_R: 5′ TTCTGGGTCCCCTCCTGAGGA 3′.

### Cytokine Measurement

Cytokine level was measured using Milliplex MAP Human Cytokine/Chemokine Magnetic Bead panels (Millipore). A 10-plex kit for IFNγ, IL-10, IL-1β, IL-6, TNFα, IL-12(P40), IL-17, IL-2, IL-4, and IP-10, a 3-plex kit for IL-8, IL-15 and MIP1A (Cat N. HCYTOMAG-60K) and a 1-plex kit for GranzymeB (Cat. N. HCD8MAG-15K) were used. The assay was performed following manufacturer's instructions. Samples were run in a Luminex 200 system.

Alternatively, intracellular cytokine production (IFNγ and TNFα) was measured by flow cytometry. Briefly, cells were first labeled for extracellular markers CD4, CD8, and CD3 and incubated 15 min. Cells were then fixed using 1X BD lysing solution (Cat. N. 349202) and incubated for an additional 15 min. After 2 washes, cells were permeabilized using FACS buffer + 0.1% saponin, and incubated for 15 min. Cells were then incubated with anti-IFNγ and anti-TNFα, for 30 min at 4°C. After that, cells were washed in PBS and analyzed.

### Small-Scale T Cell Expansions

0.5 × 10^6^ T-cells were cultured with X-Vivo 15 Cell Medium (Cultek, Cat. N. BE02-060Q), 5% AB human serum (Sigma, Cat. N. H4522), penicillin-streptomycin (100 μg/ml) and the indicated cytokine: 50 IU/ml IL-2 (Miltenyi Biotec) or 155 IU/mL IL-7 and 290 IU/mL IL-15 (Miltenyi Biotec). Cytokines were added to the media every 48 h. Twenty-seven hours after thawing cells were activated with Dynabeads Human T-Activator CD3/CD28 (Gibco, Cat. N. 11131D) according to the manufacturer's instructions. Cells were transduced after an additional 24 h with an MOI of 10 and then expanded for 11 days at a concentration of 0.5 × 10^6^ to 1.5 × 10^6^ T-cells/ml.

### T-Cell Expansion After Repeated Challenges With Target Cells

To analyze T-cell proliferation capacity after antigen encounter, we seeded a co-culture of CAR-T cells and NALM6 cells at 1:1 ratio (250,000 cells each). After 4 days of incubation, an aliquot of the culture was taken and analyzed to determine T-cell number. Cells were labeled with CD3, CD4, CD8, and CD19, and then 20 μl of beads (CountBright, Cat. N. C36950, Invitrogen) was added to the sample to determine absolute cell number. This process was repeated 3 times.

### Statistics

Statistical significance was assessed using SPSS software. Unpaired T-test was used unless otherwise specified. U-Mann Whitney was used for comparison of variables with non-normal distributions. Statistical significance was considered when *p*-value ≤ 0.05.

## Results

### CAR T-Cell Expansion

Twenty-eight apheresis products were obtained from 27 patients included in the clinical trial. For one patient, the apheresis product was obtained twice due to ARI-0001 cell production failure (T10 and T13 products belong to the same patient). Description of apheresis products is presented in Table 2. Patients' apheresis products were subjected to CD4+ and CD8+ magnetic selection using the CliniMACS Prodigy system. In all cases except for one (Patient T27), the minimum number of T-cells (100 × 10^6^) was obtained (Table 2). In Patient T27, cell culture was initiated with 50 × 10^6^ cells.

**Table 2:** Apheresis products composition and CD4-CD8 cell selection.

**Pat. ID**	**Disease**	**Apheresis products**					**CD4-CD8 selection**
		**WBC (×10^**9**^)**	**Lymph (×10^**9**^)**	CD3+ (%)^*^	CD4+ (%)^*^	CD8+ (%)^*^	**Total cell number (×10^**6**^)**
T01	Adult ALL	7.15	6.40	24.80	12.60	10.70	960
T02	NHL	3.31	1.56	32.50	11.40	19.80	1,200
T03	Pediatric ALL	4.59	3.50	49.38	22.67	26.71	1,580
T04	NHL	4.04	0.85	13.50	6.60	6.20	488
T05	NHL	7.71	4.23	30.00	12.10	15.50	1,800
T06	Adult ALL	3.64	3.19	28.80	10.60	16.20	720
T07	Adult ALL	4.91	3.84	29.00	4.40	19.80	1,900
T08	CLL	2.88	2.30	19.70	8.80	10.10	1,560
T09	Pediatric ALL	4.27	3.24	21.50	11.30	8.80	1,442
T10	Adult ALL	1.00	0.72	15.30	7.90	6.10	350
T11	Adult ALL	2.08	0.98	13.90	7.90	4.40	360
T12	Pediatric ALL	8.40	6.21	2.20	1.70	0.50	150
T13	Adult ALL	1.29	0.64	26.90	8.40	15.60	250
T14	Adult ALL	3.22	2.31	38.90	17.80	18.00	2,160
T15	Adult ALL	2.88	1.28	26.10	8.70	14.50	1,050
T16	Pediatric ALL	1.98	1.22	22.60	4.80	15.90	400
T17	Adult ALL	8.55	2.82	27.90	7.40	19.20	1,400
T19	Pediatric ALL	1.09	0.20	22.50	12.80	7.50	250
T20	Adult ALL	3.73	2.23	28.60	7.20	20.70	760
T21	Pediatric ALL	4.50	2.20	36.20	18.60	14.20	400
T22	Adult ALL	8.37	3.16	42.00	21.40	19.50	750
T24	Adult ALL	25.85	22.00	4.10	1.20	0.70	200
T25	Adult ALL	0.83	0.46	32.50	5.50	25.70	1,520
T26	Pediatric ALL	0.84	0.51	6.50	1.40	4.60	200
T27	Adult ALL	0.33	0.10	10.00	3.30	6.10	50
T30	Adult ALL	2.06	1.48	46.50	13.20	28.80	871
T32	Adult ALL	2.23	0.69	52.00	19.30	27.40	2,000
T34	NHL	6.71	3.54	23.50	12.60	9.70	993

* *% of cells over WBC*.

**Figure 1:**
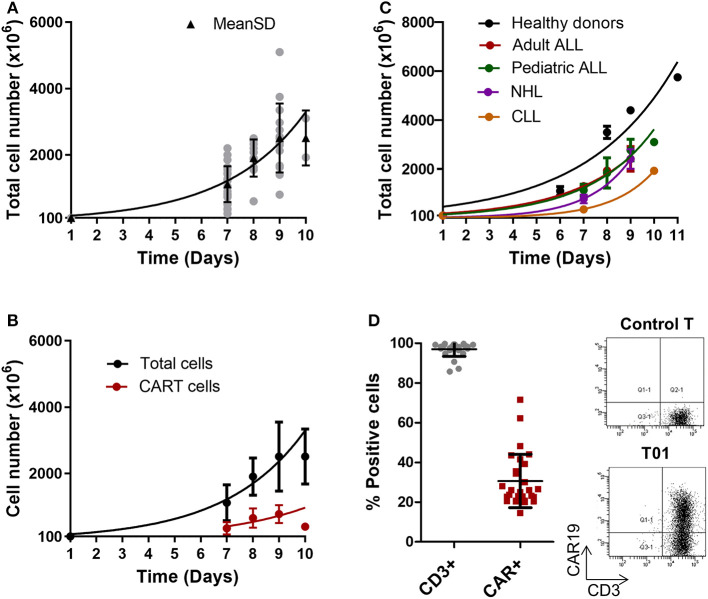
ARI-0001-cell expansion in CliniMACS Prodigy. **(A)** Expansion kinetics of ARI-0001-cell products (Total cell number). Gray points indicate individual products. Black triangles indicate Mean ± SD and adjusting curve. **(B)** Expansion kinetics of CAR19+ cells (red) and total cell number (black). Mean ± SD is represented. **(C)** Expansion kinetics of ARI-0001 cells (Total cell number) comparing healthy controls and different types of disease. Mean ± SEM is represented. **(D)** Percentage of CD3 and CAR19 positive cells as determined by flow cytometry. Mean ± SD is also indicated. Panels on the right show flow cytometry representative image corresponding to CAR19 and CD3 staining in ARI-0001-cell final products and Control T cells (Untransduced).

Results of cell expansion in CliniMACS Prodigy for the 27 products are presented in Figures 1A,B and Table S2. Cells were expanded for an average of 8.5 days, range 7–10. Average total cell number obtained in the final product was 2,548 × 10^6^, range 600 × 10^6^ to 5,200 × 10^6^. In one patient where cell culture was started with 50 × 10^6^ cells, the final product also met acceptance criteria. In this particular case, cell culture was maintained for 13 days, finally obtaining 3,300 × 10^6^ cells. When compared to healthy donors (used in three previous validation runs), patient cells seem to expand more slowly, even if the number of runs performed with healthy donors is limited (Figure 1C).

Products were analyzed in terms of appearance, quantity, identity, purity, safety, and potency. A complete list of product specifications is provided in Table S1.

### Product Purity and Transduction Efficiency

The final product was characterized in terms of cell viability, percentage of CD3+ cells and percentage of CAR+ cells. This data is summarized in Table 3. All products met acceptance criteria for cell viability and percentage of CD3+ cells (>70% for both parameters). The lowest value detected for cell viability was 91 and 85.7% for CD3+ cells (Figure 1D).

**Table 3:** ARI-0001 product characterization.

**Pat. ID**	**Cell viability**	**%CD3+**	**%CAR+**	**Sterility**	**Mycoplasma**	**Endotoxin**	**Adventitious virus**	**Transgene copies/cell**	**RCL**	**Potency**
T01	100	94.7	39.3	Sterile	Absent	<0.5 EU/ml	Absent	1.05	Absent	13.9
T02	95	96.5	28	Sterile	Absent	<0.5 EU/ml	Absent	0.92	Absent	6.0
T03	90	96.0	30	Sterile	Absent	<0.5 EU/ml	Absent	0.97	Absent	2.7
T04	98	98.1	22.8	Sterile	Absent	<0.5 EU/ml	Absent	0.66	Absent	17.2
T05	93	98.4	22.9	Sterile	Absent	<0.5 EU/ml	Absent	0.9	Absent	41.4
T06	96	87.1	43.6	Sterile	Absent	<0.5 EU/ml	Absent	1.55	Absent	0.6
T07	96	97.3	20.4	Sterile	Absent	<0.5 EU/ml	Absent	0.73	Absent	18.6
T08	92	98.7	20.7	Sterile	Absent	<0.5 EU/ml	Absent	1.62	Absent	21.4
T09	100	99.1	20.6	Sterile	Absent	<0.5 EU/ml	Absent	0.77	Absent	8.7
T10	98	98.7	14.5^*^	–	–	–	–	–	–	–
T11	94	99.7	25	Sterile	Absent	<0.5 EU/ml	Absent	2.31	Absent	0.04
T12	98	99.4	20	Sterile	Absent	<0.5 EU/ml	Absent	0.43	Absent	14.4
T13	95	99.2	20.4	Sterile	Absent	<0.5 EU/ml	Absent	0.57	Absent	3.5
T14	98	99.2	62.2	Sterile	Absent	<0.5 EU/ml	Absent	1.99	Absent	0.01
T15	93	97.5	34.4	Sterile	Absent	<0.5 EU/ml	Absent	1.98	Absent	1.4
T16	91	98.7	26.6	Sterile	Absent	<0.5 EU/ml	Absent	1.46	Absent	0.1
T17	96	94.8	26	Sterile	Absent	<0.5 EU/ml	Absent	1.47	Absent	0.2
T19	93	90.6	22.6	Sterile	Absent	<0.5 EU/ml	Absent	0.99	Absent	0.3
T20	94	98.3	20.8	Sterile	Absent	<0.5 EU/ml	Absent	1.15	Absent	0.02
T21	91	99.4	48.2	Sterile	Absent	<0.5 EU/ml	Absent	1.59	Absent	0.0
T22	82	97.3	44.1	Sterile	Absent	<0.5 EU/ml	Absent	2.1	Absent	0.0
T24	97	99.7	23.8	Sterile	Absent	<0.5 EU/ml	Absent	1.55	Absent	4.18
T25	94	98.2	41.5	Sterile	Absent	<0.5 EU/ml	Absent	2.92	Absent	0.06
T26	97	98.5	23	Sterile	Absent	<0.5 EU/ml	Absent	0.4	Absent	28.0
T27	98	96.5	23.2	Sterile	Absent	<0.5 EU/ml	Absent	0.62	Absent	4.3
T30	95	85.7	26	Sterile	Absent	<0.5 EU/ml	Absent	2.08	Absent	0.2
T32	97	98.2	35.6	Sterile	Absent	<0.5 EU/ml	Absent	1.93	Absent	0.2
T34	94	99.1	71.5	Sterile	Absent	<0.5 EU/ml	Absent	2.93	Absent	0.02

* *Value below acceptance criteria*.

To analyze the percentage of CAR+ cells, we first validated our detection method based on the use of an APC-conjugated F(ab')_2_ anti-mouse antibody. To this end, we engineered a vector where CAR19 and GFP were co-expressed. As shown in Figure S2, the correlation between GFP+APC+ or GFP–APC– cells was of 93.5%, thereby indicating that the detection method had a good sensitivity and specificity.

Using this detection system, we assessed the percentage of CAR+ (ARI-0001) cells in the patients' products. All products except one met the specification of >20% ARI-0001 cells. In one product (T10) only 14.5% ARI-0001 cells were detected. Consequently, this product was considered a production failure. CAR T-cell production was repeated for this patient from a 2nd apheresis (T13). This time, a valid product could be obtained. Mean (±SD) of percentage of CAR+ cells in this series was 30.6 ± 13.44 (Figures 1B–D), slightly lower than transduction efficiencies achieved in small-scale expansions (45.3%) (Figure S1A). No significant differences in efficiency of transduction were observed between healthy donors and patients (35.8 vs. 30.6%), or among the different diseases (Figure S1B). Percentage of CAR+ cells over time during cell expansion was also investigated. A high degree of variability was detected among patients, with the percentage of CAR+ cells increasing in some patients while decreasing in others (Figure S3). In terms of number of cell doses obtained per patient, considering a standard weight of 70 kg for adults and 25 kg for pediatric patients, a minimum of two cell doses were quickly obtained (by day 7) for all ALL patients (dose 1 × 10^6^ ARI-0001 cells/kg). For NHL patients (dose 5 × 10^6^ ARI-0001 cells/kg), two cell doses were obtained for three out of four patients, by day 9. Indeed, the number of cell doses obtained for ALL far exceeded the need (nine cell doses for adult patients and 25.4 for pediatric patients), indicating that the time of *ex vivo* cell expansion could be reduced if necessary in these groups of patients. For NHL, the average number of ARI-0001 cell doses obtained was 2.5. Only one CLL patient has been produced so far. T-cells from this patient grew slower and required 10 days of expansion, finally obtaining 398 × 10^6^ ARI-0001 cells.

CAR19 transduction was also assessed in terms of DNA copies/cell. As shown in Table 3 CAR19 was detected in all products, within a range of 0.4–2.9 copies/cell (all below the limit considered safe of <10 copies/cell). As expected, a positive correlation between percentage of CAR+ cells and DNA copies/cell was obtained, further validating both techniques (Figure S4).

### Product Potency

**Figure 2:**
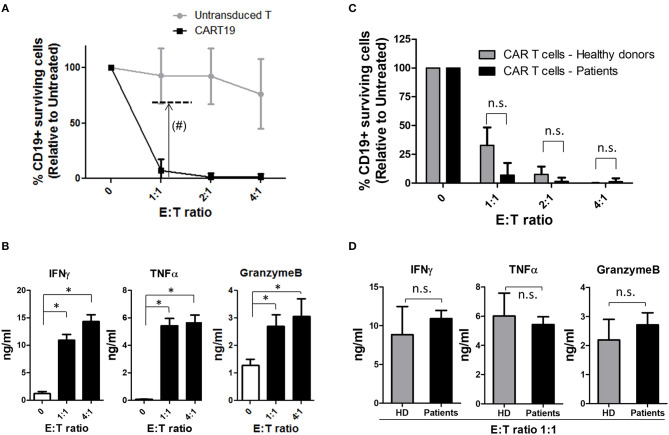
ARI-0001-cell potency. **(A)** Cytotoxicity assay after 4 h of ARI-0001 co-culture with NALM6 cells, at the indicated ratios. Mean ± SD of all 27 CAR T cell products is indicated. (#) Dashed line indicates minimum of ARI-0001-cell cytotoxicity level for a product to be considered valid. **(B)** IFNγ, TNFα, and GranzymeB levels measured in the supernatants of the cytotoxicity assays. E:T ratio 0 indicates no target cells. (*) indicates statistical significance, *p* < 0.05. **(C)** Comparison of ARI-0001 cytotoxic potential after 4 h of co-culture with NALM6 cells, at the indicated ratios. Mean ± SD is shown. “n.s.” indicates not statistically significant (Non parametric test). **(D)** Comparison of IFNγ, TNFα, and GranzymeB levels measured in the supernatants of the cytotoxicity assay at E:T ratio 1:1. “HD” indicates Healthy donors. “n.s.” indicates not statistically significant (Parametric test applied to IFNγ and TNFα and non-parametric test applied to GranzymeB).

Cytotoxic potential was analyzed *in vitro* for each product before infusion. A co-culture of the final product with NALM6 cell line was initiated at different E:T ratios. Percentage of alive-CD19+ cells was measured by flow cytometry after 4 h. As a control, the cytotoxic activity of non-transduced CD4+CD8+ cells from the same patient was also measured. Products were considered valid when the CD19+ cell surviving fraction with ARI-0001 cells, at ratio 1:1, was lower than 70%. Results are presented in Table 3 and Figure 2A. All products obtained met the specification of <70% CD19+ surviving fraction at E:T ratio 1:1, indicating that all products prepared had the intrinsic capacity of eliminating CD19+ cells.

Cytokine level was also measured in the supernatant of cytotoxicity assays. As expected, increased levels of pro-inflammatory cytokines such as IFNγ and TNFα was observed when ARI-0001 cells were co-cultured with NALM6, compared to ARI-0001 cells alone. The level of GranzymeB was also significantly increased (Figure 2B) consistent with the cytotoxic activity of ARI-0001 cells. The complete set of analyzed cytokines is provided in Figure S5.

CAR T-cells produced from patients were compared to those obtained from healthy controls in terms of cytotoxic activity and cytokine production. As shown in Figure 2C, patients' and healthy donors' CAR T-cells showed similar cytotoxic potential (even slightly higher for patient's cells although this was not statistically significant). Production of pro-inflammatory cytokines (IFNγ and TNFα) and GranzymeB was also comparable (Figure 2D).

### T Cell Subset Characterization

**Figure 3:**
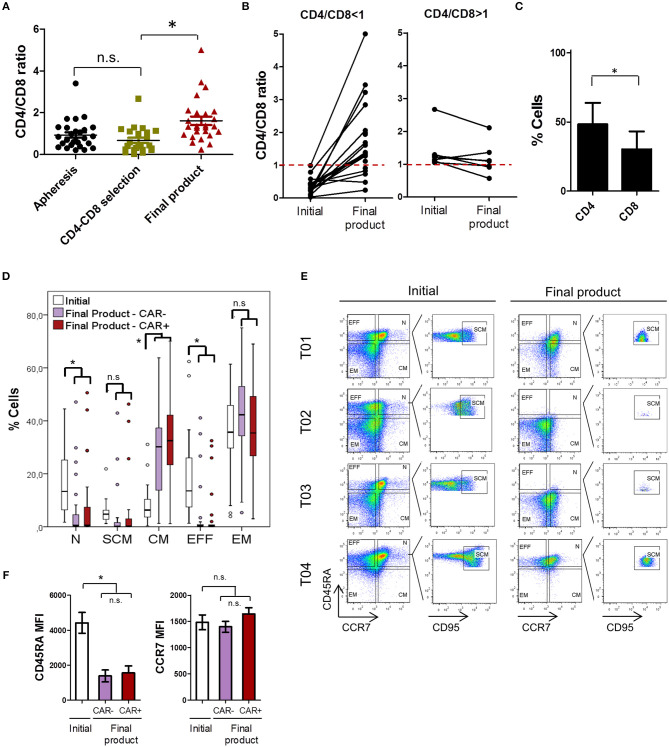
ARI-0001 cell subset characterization. **(A)** CD4/CD8 ratio of apheresis products, after CD4-CD8 cell selection and of the final product. **(B)** CD4/CD8 ratio variation during cell expansion. Left panel corresponds to products with an initial ratio < 1. Right panel corresponds to products with an initial ratio > 1. **(C)** CAR19 transduction efficiency in CD4 and CD8 cells. Mean ± SD is shown. **(D)** Percentage of T-cell subpopulations within initial (CD4-CD8 cell selection) and final products (CAR– and CAR+ cells). **(E)** Representative flow cytometry plots of three different patients showing T cell populations in initial and final products. **(F)** Differences in MFI for CD45RA and CCR7 in initial and final products. Lower panel shows paired analysis for CCR7 MFI. (*) indicates statistical significance, *p* < 0.05. n.s. indicates not statistically significant.

Product composition was further analyzed in terms of CD4/CD8 ratio and T_N_, T_SCM_, T_CM_, T_E_, and T_EM_ subsets. Consistent with previous reports (38), CD4/CD8 ratio was inverted (CD4/CD8 ratio <1) in a large subset of patients that were candidate for a CAR T-cell therapy (Figure 3A). Average CD4/CD8 ratio was 0.93 ± 0.67 in the apheresis products. This ratio was not significantly altered after CD4 and CD8 cell selection in the vast majority of patients. However, a significant increase in the proportion of CD4 cells was detected during cell expansion. CD4/CD8 ratio increased from 0.64 ± 0.61 after CD4-CD8 cell selection, to 1.61 ± 1.04 in the final product. A deeper analysis of this data reveled that in patients starting with a CD4/CD8 ratio <1, the proportion of CD4+ cells tended to increase during cell expansion, while in patients where a CD4/CD8 ratio > 1 was obtained after cell selection, the proportion of CD4+ cells tended to decrease (Figure 3B). Therefore, the difference in CD4/CD8 ratio (ΔCD4/CD8) before and after cell expansion was significantly different depending on the initial ratio (Figure S6A). The efficiency of transduction differed between CD4+ and CD8+ subsets, as CD4 showed a significantly higher percentage of CAR+ cells (Figure 3C).

In terms of T_N_, T_SCM_, T_CM_, T_E_, and T_EM_ subsets, we observed a high degree of variability among patients' final products (Figure 3D). This high variability is exemplified by the different level of CD45RA and CCR7 expression in samples from different patients (Figures 3E,F) and cannot be attributed to the different diseases (Figure S1C). Within the CAR+ T-cells of the final product, memory phenotypes (CM and EM) predominated in the vast majority of patients. Average percentage and SD for each subpopulation in the CAR+ cells of the final product is as follows: T_N_: 7.71 ± 13.9, T_SCM_: 5.26 ± 12.0, T_CM_: 31.01 ± 16.7, T_EM_: 35.11 ± 17.7, and T_E_: 4.2 ± 9.5. Analysis of CD4 and CD8 cells separately showed that CD8 cells have a more T_N_, T_SCM_, and T_CM_ phenotype than CD4 cells (Figure S6B). We also analyzed how these subsets varied during *ex vivo* cell expansion by comparing T-cell subsets in the initial (after CD4-CD8 cell selection) and in the final product, and if CAR expression influenced T-cell subpopulations (CAR– vs. CAR+ cells). As shown in Figure 3D, we observed a robust increase in the proportion of T_CM_ during cell expansion while T_N_ and T_EFF_ cells decreased. These changes in T-cell subsets can be attributed to a decrease in CD45RA expression which is expected upon cell activation (Figures 3E,F).

No statistically significant changes in the T-cell subsets were detected between CAR– and CAR+ cells in the final product, although a further increase in T_CM_, and consequent decrease in T_EFF_ cells, was observed in CAR+ cells compared to CAR– (Figure 3D). Consistently, a small increase in CCR7 expression was also detected in CAR+ cells vs. CAR- cells. (Figure 3F). The impact of CAR expression on CCR7 was further explored in independent small-scale expansions (see next section). The changes in expression of CD27, CD28, and CD95 were also assessed by flow cytometry. As shown in Figure S6C, CD95 was increased during cell expansion and CD27 decreased. CD28 did not show significant changes during expansion, although presented a higher expression in CAR+ compared to CAR- cells.

### Small-Scale CAR T Cell Expansions

To further evaluate the impact of culture conditions or CAR expression on the proportion of CD4/CD8 ratio or T-cell phenotype, cell expansions from patients' selected cells were repeated in a small scale experiment, under different conditions. We selected six of the patients (three adult ALL and three NHL) from which frozen leftover cells after CD4-CD8 cell selection were available. We expanded patients' cells in four different conditions: (1a) IL2—Untransduced T-cells, (1b) IL2—CAR T-cells, (2a) IL7/IL15—Untransduced T-cells, (2b) IL7/IL15—CAR T-cells. As shown in Figure S7A, cells expanded between 17 and 100 times over a period of 11 days. CAR19 transduced T-cells expanded less (or slowly) compared to untransduced counterparts, and IL2 grown cells expanded more than IL7/IL15 (in both untransduced and CAR19 conditions) (Figure S7B). Cell transduction or cytokines used did not condition CD4/CD8 ratio in a consistent way. However, as detected previously in the products expanded using the Prodigy system, in patients starting with CD4/CD8 ratio>1 (T04 and T34), the ratio tended to decrease, while in patients starting with CD4/CD8 ratio <1 (T02, T15, T22, and T34), the ratio tended to increase (Figure S7C). Indeed, since the expansions were maintained for longer in the small-scale expansions than in the Prodigy system, we observed that the ratio CD4/CD8 may fluctuate in a more or less pronounced way, but it tends to CD4/CD8 = 1 if the cells are cultured for longer periods of time.

Interestingly, significant differences were found in terms of T-cell subsets depending on the culture conditions. The cytokines used in the growth media did not provide significant differences in terms of the different subsets in this series of patients. However, a significant and consistent difference was appreciated in CAR19 expressing cells vs. untransduced T-cells for almost all subsets. As shown in Figure S7D, CAR19 transduction resulted in a much higher percentage of T_N_, T_SCM_ and T_CM_ subsets independently of the cytokine used in the culture media. By contrary, T_EM_ cells were decreased in the CAR19+ cells compared to the untransduced samples. In this case, no difference in CD45RA MFI between untransduced and CAR19+ cells was observed that can account for the decrease in T_N_ and T_SCM_, since in both conditions, cells were activated and proliferated *ex vivo*. However, we observed a significant increase in CCR7 expression in CAR19+ cells compared to untransduced cells (Figure S7E). This increase explains a higher percentage of T_N_, T_SCM_, and T_CM_ subsets and lower T_EM_. Increase in CCR7 expression upon 4-1BB activation has been previously described in monocytes (39) and also proposed for CAR T-cells (40). To test if 4-1BB activation is responsible for the increase in CCR7 we observe in the CAR+ cells, we modified our CAR construct by changing the co-stimulatory domain to CD28 (Figure S8A). T-cells from a healthy donor were then left untransduced or transduced with the 4-1BB- or CD28-containing CARs and expanded *in vitro* for 10 days. Again, we observed an increase in CCR7 expression in the CAR-positive fraction of the cells transduced with the 4-1BB-containing construct, compared to untransduced cells or CD28-containing CAR+ cells (Figure S8B). As expected, percentage of T_CM_ cells is also higher in 4-1BB-containing CAR+ cells (Figure S8C).

Finally, the functionality of CAR T-cells manufactured with the Prodigy system and small-scale expansions was also compared. For this comparison, cells from 3 patients expanded with IL-7/IL-15 were used. The production of pro-inflammatory cytokines, cytotoxic potential and T-cell expansion was measured after adjusting for the same percentage of CAR+ cells. Production of IFNγ and TNFα was measured after co-culture of CAR T-cells with NALM6 at 1:1 ratio, at 4 h time-point. Level of these two cytokines was measured both by intracellular staining (Figure S9A) and cytokines present in the media (Figure S9B), yielding consistent results. Cells manufactured in the Prodigy system consistently produced slightly more IFNγ and TNFα than cells manufactured in small-scale expansions. However, these differences were not statistically significant. In terms of cytotoxic potential, cells produced with both methods showed comparable results (Figure S9C). Finally, T-cell expansion upon repeated challenges with fresh target cells (NALM6) was slightly higher in cells manufactured with the Prodigy system than with small-scale expansions, although it did not reach statistical significance (Figure S9D). Therefore, we conclude that cells manufactured with the Prodigy system are functionally comparable, or even slightly more active, than those produced in small-scale expansions.

Taking all this data together, we conclude that *ex vivo* cell expansion causes a loss of T_N_ and T_EFF_, which is observed in both the Prodigy system and small-scale expansions. On the contrary, T_CM_ cells are largely accumulated, both by *ex vivo* expansion and as a result of CAR expression (in CARs containing 4-1BB as a co-stimulatory domain). Cells produced in Prodigy system are functionally similar to those produced in small-scale expansions.

## Discussion

CAR T-cells are complex medical products. Most of the approaches explored so far are based on the use of autologous CAR T-cells, thereby requiring the production of a personalized product for each patient. Moreover, CAR T-cells are gene-therapy products, involving clinical-grade preparation of vectors, and subsequent T-cell selection, transduction and expansion. Two different CAR T-products (Yescarta, Kite-Gilead and Kymriah, Novartis) have already been approved for the treatment of pediatric-ALL and NHL. Nonetheless, the development of new CAR T-products, including phase I and II clinical trials, for many different targets and diseases is primarily conducted in academic research centers. Manufacturing of clinical-grade CAR T-cells can pose a challenge for many medium-sized academic institutions. We have previously published the development of our own CAR19 product (based on the scFv sequence of A3B1 antibody) and the establishment of a CAR T-cell production protocol and infrastructure, based on the use of CliniMACS Prodigy, a semi-automated closed system by Miltenyi (37). We and others have previously demonstrated the feasibility of CAR T-cell manufacturing from healthy donors, using CliniMACS Prodigy (41–45). Now, we report the preparation and characterization of 27 CAR T-cell products for ALL, CLL, or NHL patients.

Patients eligible for CAR T-cell therapies have previously received multiple lines of therapies. As a result, T-cells from these patients have a reduced proliferation capacity and abnormal CD4/CD8 ratio compared to healthy donors (46, 47). Nevertheless, we have been able to obtain 27 valid CAR T-cell products (according to the specifications indicated in Table S1) out of 28 attempts, proving the feasibility of CAR T-cell manufacturing for heavily pre-treated patients using the Prodigy system. Cell expansion was maintained for an average of 8.5 days. Indeed, patient-derived T-cells expand more slowly compared to the expansions performed from healthy T-cell donors. However, for ALL patients, where the infused dose is typically 1 × 10^6^ CAR T-cells/kg, a minimum of 2 CAR T-cell doses were already achieved by day 7 in a 100% of patients, indicating that it is possible to reduce the expansion time in this subset of patients (actual expansion time was 8.4 days, due to pre-scheduled work plans). CLL and NHL patients required 9–10 days of expansion due to reduced intrinsic T-cell proliferation (48, 49) and higher CAR T-cell numbers needed, respectively. Reduced T-cell expansion time is highly desirable, not only to reduce the cost of batch production and to shorten the time until the patient can receive the treatment, but also for the quality of the product obtained, as discussed below. Despite reduced expansion capacity of heavily pretreated patient's T-cells compared to healthy donors, we have observed equivalent product potency in terms of cytotoxic potential and pro-inflammatory cytokine production. The method of CAR T-cell production (Prodigy vs. small-scale) yielded also comparable products in terms of product potency, or even slightly more active in cells obtained with Prodigy. We reasoned that the higher cell densities reached in the Prodigy system may be advantageous for T-cell fitness and potency.

Average transduction efficiency was 30.6% on average in our series and does not differ significantly between patients and healthy controls. Similar transduction efficiencies have been reported by other groups using CliniMACS Prodigy for production of CAR T-cells from healthy controls (41, 50–52), except one study that reports transduction efficiencies between 50–60% using very high MOI (MOI = 100) (44). Transduction efficiency in small-scale CAR-T cell productions is slightly higher (45.3 vs. 30.6%). We can speculate that having the possibility to put in direct contact cells and lentivirus, i.e., spinoculation, or adding reagents to the media that facilitate the entry of the lentivirus to the cells (such as polybrene) could help increase transduction efficiency in the Prodigy system.

We observed a preference of CD4+ cell expansion over CD8+ *ex vivo*. This is consistent with previous observations from other groups using different expansion systems and CAR constructs (53, 54). Gomes-Silva and co-workers reported a preferential expansion of CD4 cells when using a 4-1BB co-stimulatory domain compared to a CD28-containing CAR (55). Therefore, this effect may contribute partially to explain this phenomenon. Indeed, we detected a higher fraction of CD4-transduced cells than CD8. Nevertheless, we have clearly shown than CD4/CD8 ratios are highly dependent on the initial ratio and tend to equilibrium in longer *ex vivo* expansions, indicating that preferential CD4 expansion may be transitory and fluctuate also after CAR T-cell infusion.

T_N_, T_SCM_, and T_CM_ phenotypes have been associated to longer persistence *in vivo* and higher anti-tumor efficacy (38, 56–58). We have dissected the factors that determine these T-cell populations with the aim of analyzing if our production system could be optimized. Indeed, there is a very high degree of variability in T-cell phenotypes among patients, which highly determines final product composition. In our experimental setting, both IL-7/IL-15 and IL-2 yielded comparable population phenotypes. In most patients, *ex vivo* cell culture led to a decrease in the fraction of CD45RA positive cells in the final product. Loss of CD45RA expression is expected upon cell activation and proliferation, and therefore intrinsic to *ex vivo* cell expansion, resulting in decreased numbers of T_N_ cells (38, 40, 59, 60). In this regard, shortening the time of *ex vivo* cell expansion when possible may help preserve this population. T_CM_ cells have also been shown to be able to establish persistent T-cell memory (58) and have superior anti-tumor effects (38). Therefore, the loss of T_N_ cells may not have a major impact in anti-tumor efficacy, since most of them are converted to T_CM_ cells. Actually, memory phenotypes are the most represented populations in our CAR T final products, consistent with previous reports (53). 4-1BB-containing CAR T-cells have been shown to have an increased proportion of T_CM_ compared to CD28-containg CARs, which have a predominant T_EM_ population (40, 55). We have shown that the increase in T_CM_ cells is not only the result of CD45RA loss, but also due to increased CCR7 expression in 4-1BB-containing CAR-expressing cells [our data and Kawalekar et al. (40)]. This data correlates with a longer CAR T-cell persistence *in vivo* of 4-1BB- compared to CD28-containing CARs (11, 12, 49).

In conclusion, we have demonstrated for the first time the feasibility of CAR T-cell manufacturing for heavily pre-treated ALL, CLL, and NHL patients, using CliniMACS Prodigy. To our knowledge, this is the first report describing the characteristics of the products obtained with this system, including a medium-large cohort of patients. Our study shows that CAR T-cell manufacturing can be completed in as low as 7 days for ALL patients and that reduced *ex vivo* expansion time may yield CAR T-cell products with increased persistence *in vivo*. The products obtained show potent anti-tumor efficacy and are characterized by a predominance of T_CM_ and T_EM_ phenotypes.

## Data Availability Statement

All datasets generated for this study are included in the article/Supplementary Material.

## Ethics Statement

This study has been approved by the Research Ethics Comitee (CeIm) of Hospital Clinic. HCB/2017/0001. Clinical trial: CART19-BE-01. Eudra: 2016-002972-29. Written informed consent to participate in this study was provided by the participants' legal guardian/next of kin.

## Author Contributions

MC designed and performed experiments, analyzed data, and wrote the manuscript. MC-B and VO-M coordinated specific parts of the clinical trial and analyzed data. EG-N, GS, AA-V, AB, BM, and LM performed experiments and procedures. BM-A, JCi, ML, EG, JT, ET, UP, JCa, TB, and DB-R supervised procedures. EC critically read the manuscript. ÁU-I, JY SR, JD, and MJ coordinated and supervised the study.

### Conflict of Interest

SR declares speakers bureau and travel expenses: Novartis, Shire, JazzPharma, Erytech. The remaining authors declare that the research was conducted in the absence of any commercial or financial relationships that could be construed as a potential conflict of interest.
