# Supplementary material for: Point-Of-Care CAR T-Cell Production (ARI-0001) Using a Closed Semi-automatic Bioreactor: Experience From an Academic Phase I Clinical Trial
Source: Front Immunol. 2020 Mar 20;11:482. doi: 10.3389/fimmu.2020.00482 (PMC7259426; doi:10.3389/fimmu.2020.00482)
Supplement: Supplementary file 1 [file Data_Sheet_1.PDF]

Supplemental information

1. Supplemental figures
2. Supplemental tables

1. Supplemental figures

Figure S1. Characterization of ARI-0001 products. Comparison of %CAR+ cells between small-scale and Prodigy manufacturing systems and analyses by type of disease

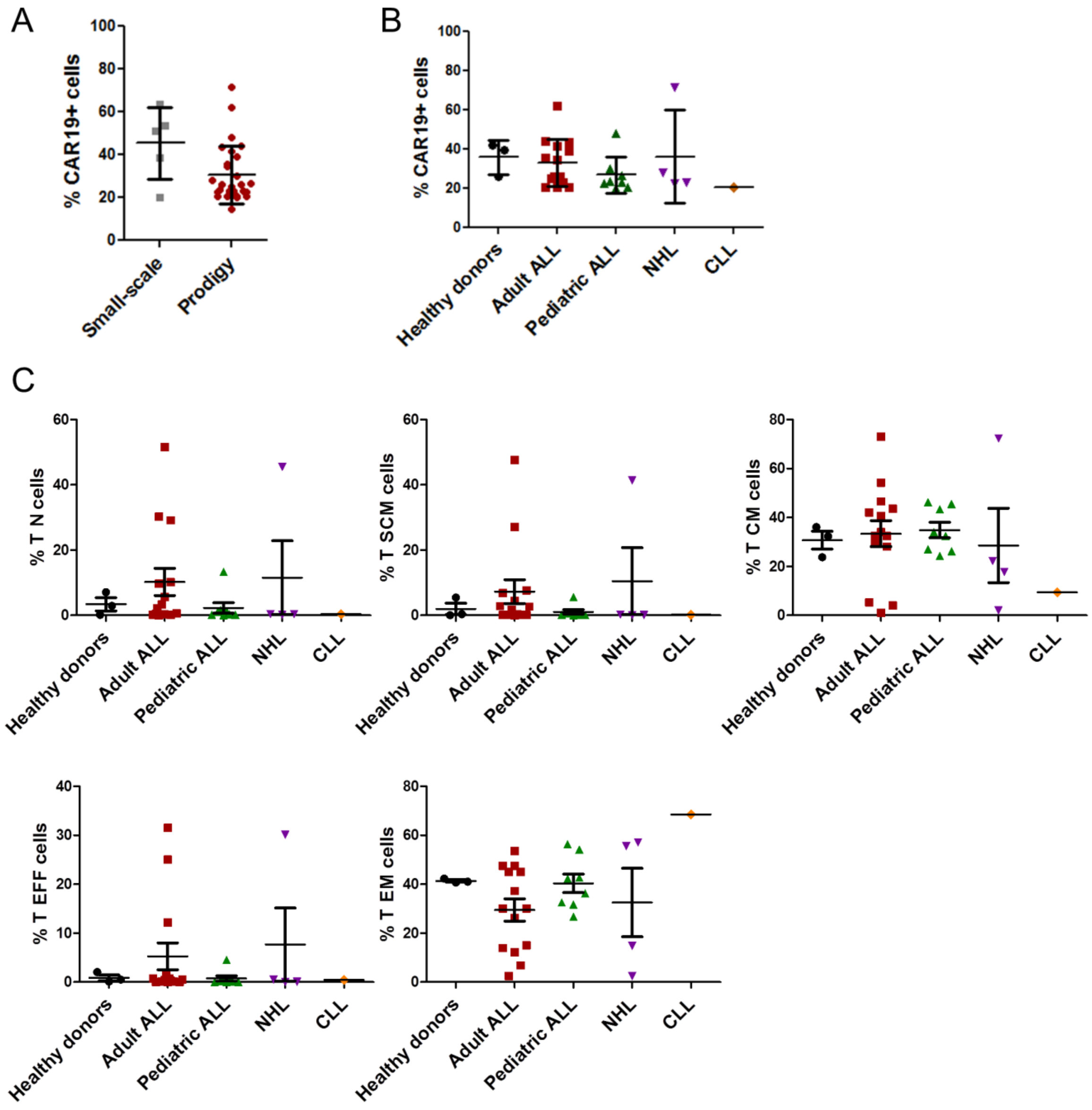

(A) Percentage of CAR+ cells comparing small-scale and Prodigy manufacturing system. Mean±SD is shown in black. (B) Percentage of CAR+ cells comparing healthy controls and different types of disease. Mean±SD is shown in black. (C) Frequency of T cell subsets in ARI-0001 final products (CAR+ cells) comparing healthy controls and different types of disease. Mean±SD is shown in black.

Figure S2. Validation of detection method for CAR19-expressing cells

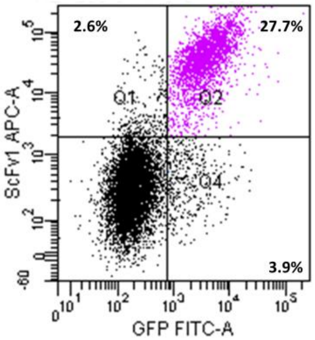

Detection of CAR-expression in GFP-co-expressing cells using an APC-conjugated $F(ab')_2$ anti-mouse antibody.

Figure S3. Variation of CAR expression during *ex vivo* cell expansion and comparison with small-scale expansions.

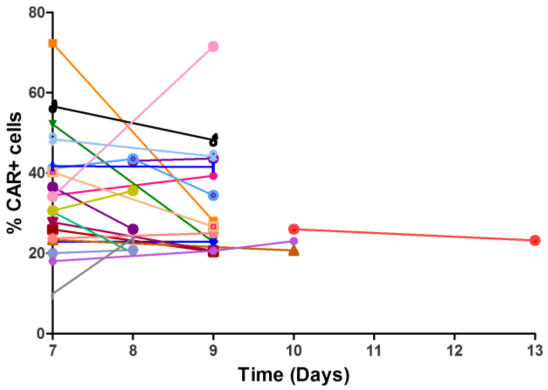

CAR expression was evaluated at different times during *ex vivo* cell expansion. Each line shows a single patient.

Figure S4. Correlation of % CAR+ cells and transgene copies/cell

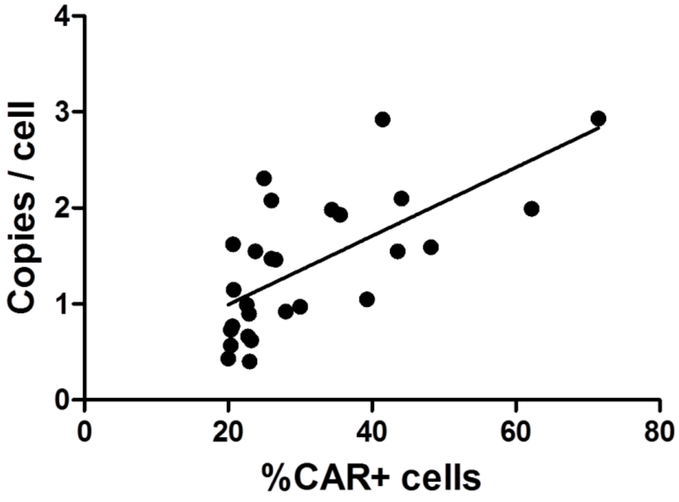

CAR expression was evaluated using 2 different techniques: (i) %CAR+ cells using flow-cytometry and (ii) Transgene copies/cell using quantitative real-time PCR. A significant positive correlation was detected between the two methods. Fisher test, p=0.0001, r^2=0.44

Figure S5. Cytokine levels from supernatants of co-culture experiments

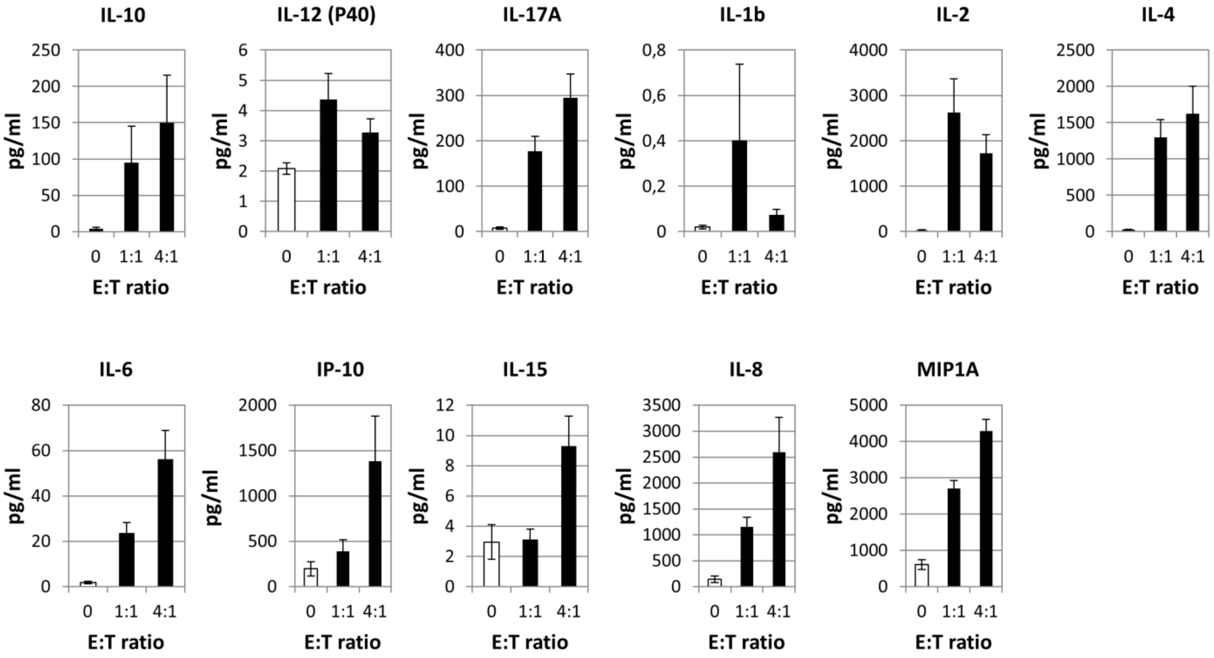

Cytokine levels measured from supernatants of co-culture experiments of final products with NALM6 cells at different E:T ratios. Cytokine levels were measured by Luminex. Graphs show mean ± SEM.

Figure S6. Characterization of T-cell subpopulations in initial and final products.

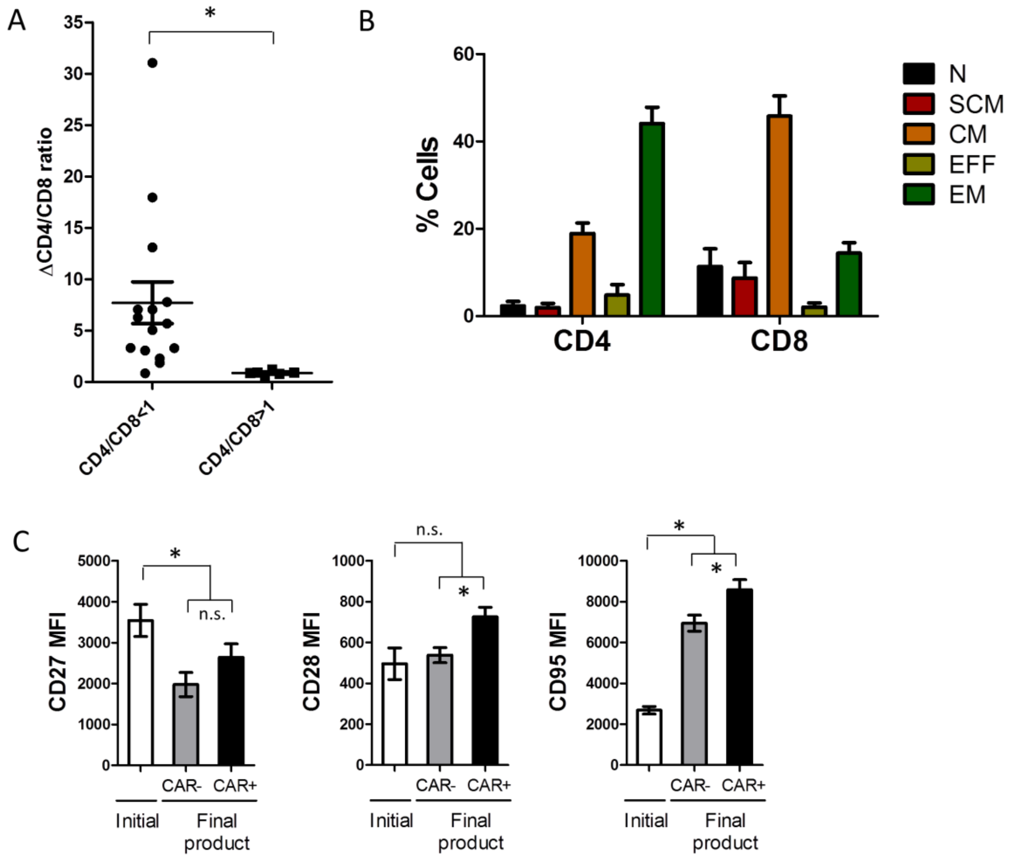

(A) Change in CD4/CD8 ratio during cell expansion (ΔCD4/CD8) depending on the initial CD4/CD8 ratio. Lines indicate mean ± SEM. (B) T-cell phenotype of CD4+CAR+ and CD8+CAR+ cells in the final product. Bars indicate mean ± SEM. (C) MFI of CD27, CD28 and CD95 in initial and final products. Bars indicate mean ± SEM. (*) indicates statistical significance ($p<0.05$). n.s. indicates no statistical significance.

Figure S7. Small-scale T-cell expansions comparing IL2 v.s. IL7/15 and untransduced v.s. CAR-expressing cells.

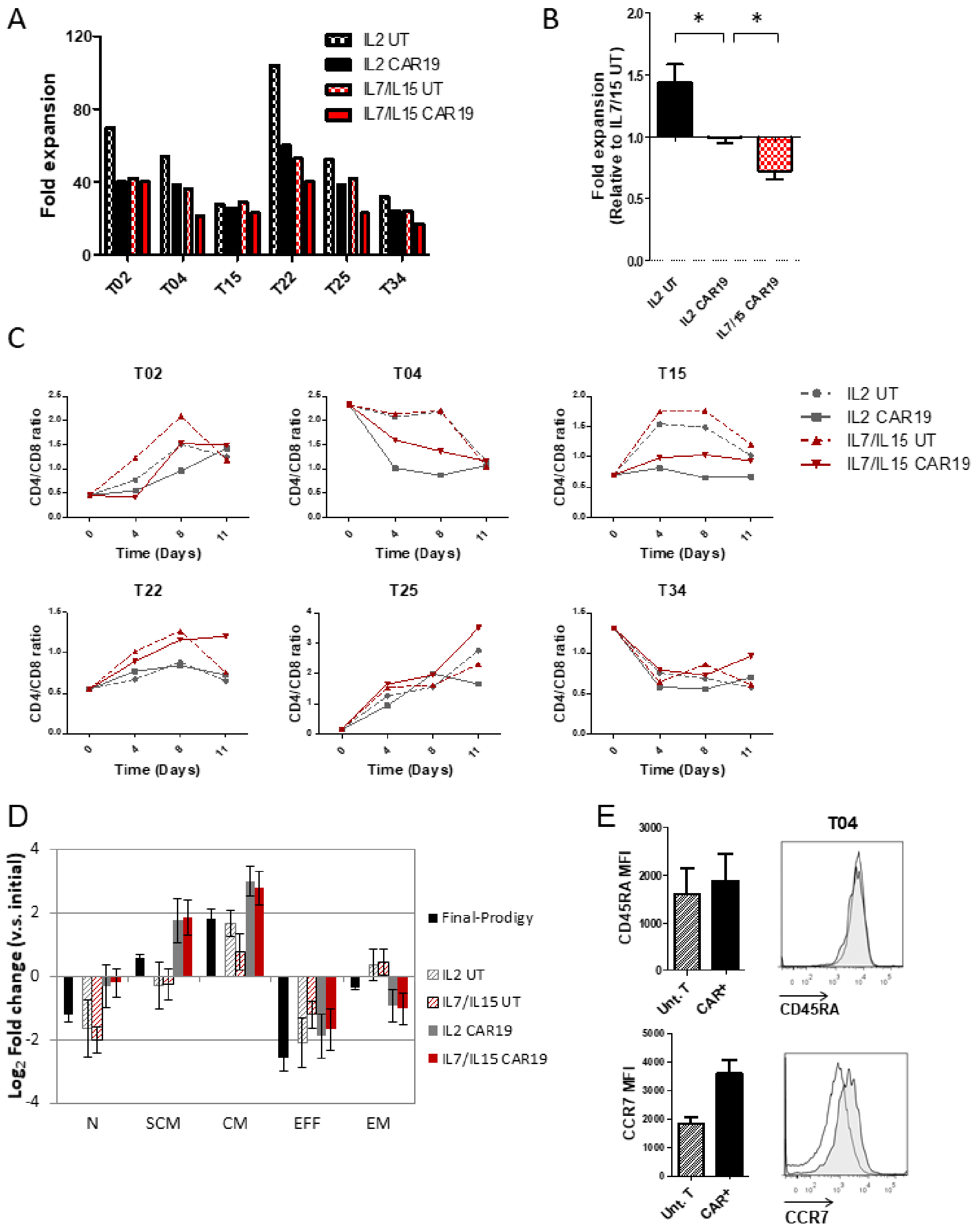

(A) Fold expansion of T-cells in the four different conditions. (B) Fold expansion of T-cells in the four different conditions relative to IL7/IL15 untransduced cells. Bars indicate mean ± SEM (C) CD4/CD8 ratio variation during cell expansions in the four different conditions. Dashed lines highlight CD4/CD8 ratio =1. (D) Change in T-cell phenotype during cell expansion, expressed as Log2 fold change. Mean of 6 patients ± SEM is shown. (E) Changes in CD45RA and CCR7 MFI between untransduced cells and CAR-expressing cells. Right panels show data corresponding to a representative patient. (*) indicates statistical significance, p>0.05. (UT) indicates untransduced.

Figure S8. CAR T cell phenotype of 4-1BB vs CD28-containing CAR.

A

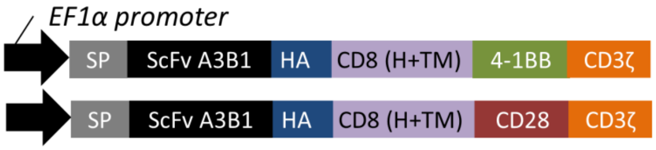

B

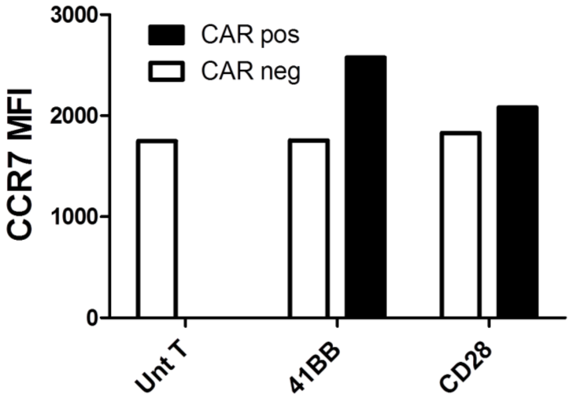

C

	Unt T	4-1BB		CD28	
		CAR-	CAR+	CAR-	CAR+
**N**	2.9	2.1	2.5	1.8	2.0
**SCM**	2.6	1.7	2.3	1.6	1.7
**CM**	34.8	37.4	42.6	36.5	40.0
**EM**	41.9	40.3	34.9	42.0	38.7
**EFF**	0.4	0.2	0.2	0.2	0.2

(A) Diagrams showing molecular domains of 4-1BB – and CD28-containing CARs. (SP) Signal Peptide, (HA) HA-tag, (H) Hinge, (TM) Transmembrane domain; (B) CCR7 expression levels (reported as MFI) in Untransduced cells, transduced with 4-1BB-containing CAR or CD28-containing CAR. CAR+ and CAR- cells have been analyzed separately for the transduced conditions. (C) Percentage of each T cell phenotype in the same T-cell populations analyzed in (B).

Figure S9. Functional comparison of CAR T-cells manufactured with Prodigy system and small-scale expansions.

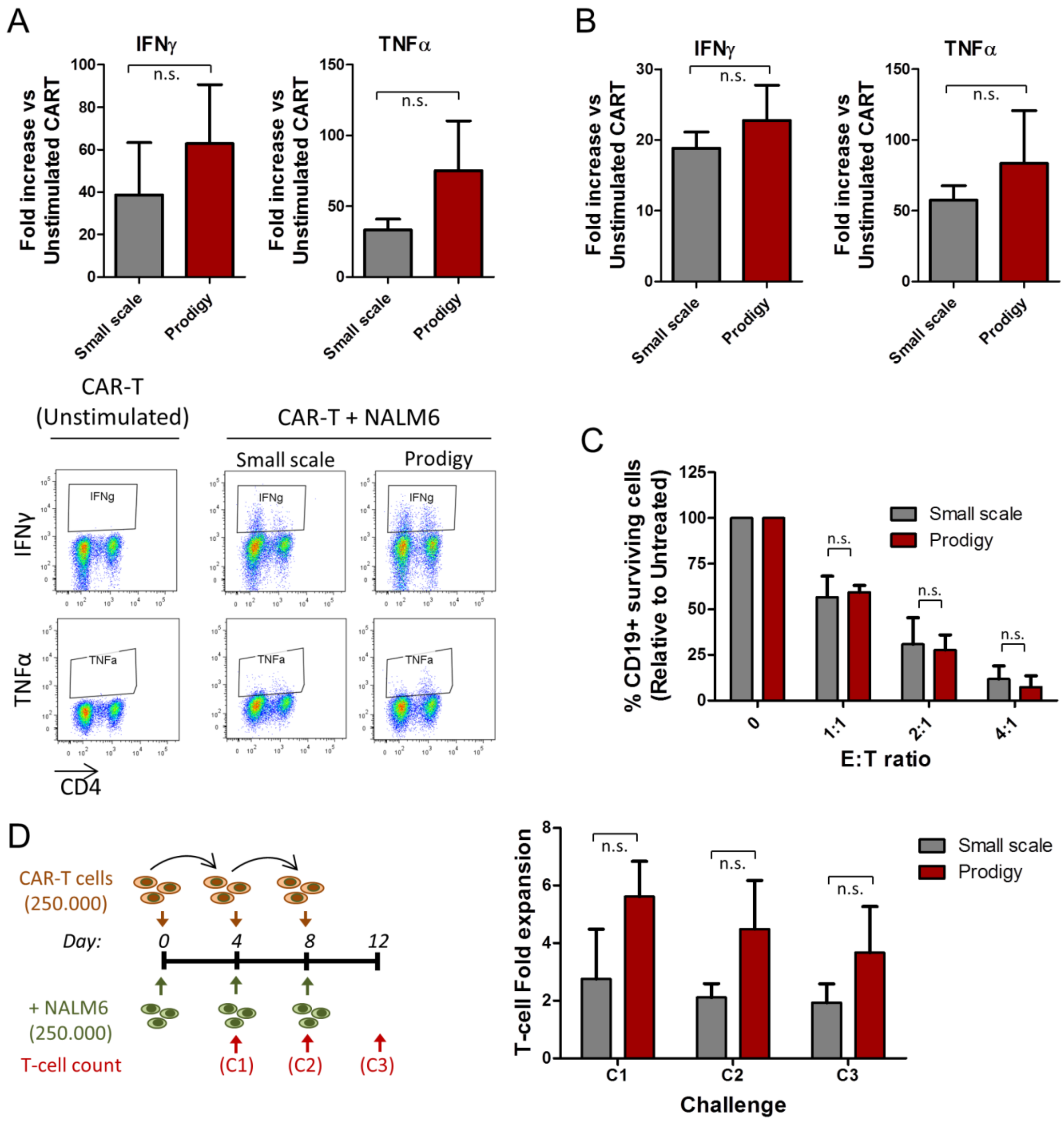

(A) Intracellular cytokine measurement using flow cytometry. Upper panels show fold increase in cytokine production over CAR T-cells alone (Unstimulated). Cytokines measured 4h after co-culture of CAR T-cells and NALM6 cells at ratio 1:1. Bars indicate mean ± SEM. “n.s.”indicates no statistical significance. Lower panels show representative images. (B) Cytokines measured by Luminex in the media of co-cultures with NALM6 cells at 1:1 ratio, at 4h time-point. Fold increase in cytokine production over CAR T-cells alone (Unstimulated) is shown. Bars indicate mean ± SEM. “n.s.”indicates no statistical significance. (C) Cytotoxicity assay of CAR T-cells and NALM6 cells at 4h time-point, at the indicated ratios. E:T ratio =0 indicates no effector cells. “n.s.”indicates no statistical significance. (D) T-cell proliferation after repeated challenges with NALM6 cells, at ratio 1:1. Every 4 days, 250.000 T-cells from the previous challenge are seeded together with fresh target cells. Left panel shows a diagram of experimental design. Right panel shows T-cell fold expansion after each challenge. Bars indicate mean ± SD. “n.s.”indicates no statistical significance.

2. Supplemental Tables

Table S1. ARI-0001 product specifications

Parameter	Method	Acceptance criteria
Appearance	Visual inspection	Cloudy liquid solution
Number of CAR19+ cells	Neubauer and flow cytometry	>0.5 x 10^6 CART cells/kg
% CAR19 cells	Flow cytometry	≥20%
% CD3	Flow cytomtery	≥70%
% Cell viability	Neubauer – trypan blue	≥70%
Sterility	Microbial growth	Sterile
Mycoplasma	PCR	Absent
Endotoxin	Chromogenic assay	≤0.5EU/ml
Adventitious virus	PCR	Absent
Transgene copies / cell	Real-time PCR	≤ 10 copies / cell
RCL	Real-time PCR	Absent
Potency	Flow cytometry	<70%*

* NALM6 cell surviving fraction using CART19 ratio E:T 1:1

Table S2. Cell expansion in CliniMACS Prodigy

Pat. ID	Total cell count (x10^6)							Days in culture
	Day 1	Day 7	Day 8	Day 9	Day 10	Day 13	Final product	
T01	100	1550	1900	2360			2630	9
T02	100	525	n.d.	2375			2375	9
T03	100	900	2500				2500	8
T04	100	500	n.d	2500			3300	9
T05	100	1275	n.d	3350			3060	9
T06	100	n.d	2075	2300			3700	9
T07	100	1625	n.d	5100			5200	9
T08	100	350	n.d	n.d	1925		1600	10
T09	100	1250	n.d	3750			4500	9
T11	100	1225	n.d	2125			2900	9
T12	100	230	600				600	8
T13	100	1425					1150	7
T14	100	n.d	1550				2100	8
T15	100	2000	2325	2550			3400	9
T16	100	825	n.d	2975			3000	9
T17	100	1625					2000	7
T19	100	1850					1280	7
T20	100	1265	1625				1520	8
T21	100	1450	n.d	2750			2340	9
T22	100	725	n.d	800			600	9
T24	100	2200	2425				3600	8
T25	100	750	n.d	1675			2400	9
T26	100	375	n.d	1625	3100		4400	10
T27*	50	n.d	n.d		1500	3300	3300	13
T30	100	1050	2200				2200	8
T32	100	1025	1875				1810	8
T34	100	800	n.d	1400			2100	9

n.d. Not determined
